# IL4-10 Fusion Protein Shows DMOAD Activity in a Rat Osteoarthritis Model

**DOI:** 10.1177/19476035211026736

**Published:** 2021-06-23

**Authors:** E.M. van Helvoort, H.M. de Visser, F.P.J.G. Lafeber, K. Coeleveld, S. Versteeg, H.H Weinans, J. Popov-Celeketic, N. Eijkelkamp, S.C. Mastbergen

**Affiliations:** 1 Department of Rheumatology & Clinical Immunology, University Medical Center Utrecht, Utrecht University, Utrecht, The Netherlands; 2 Department of Orthopaedics, University Medical Center Utrecht, Utrecht University, Utrecht, The Netherlands; 3 Center for Translational Immunology, University Medical Center Utrecht, Utrecht University, Utrecht, The Netherlands

**Keywords:** osteoarthritis, disease-modifying osteoarthritis drugs (DMOAD), animal model, pain, chondroprotection

## Abstract

**Objective:**

Ideally, disease-modifying osteoarthritis (OA) drugs (DMOAD) should combine chondroprotective, anti-inflammatory, and analgesic effects in a single molecule. A fusion protein of interleukin-4 (IL-4) and IL-10 (IL4-10 FP) possesses these combined effects. In this study, the DMOAD activity of rat IL4-10 FP (rIL4-10 FP) was tested in a rat model of surgically induced OA under metabolic dysregulation.

**Design:**

rIL4-10 FP was produced with HEK293F cells. Bioactivity of purified rIL4-10 FP was determined in a whole blood assay. Male Wistar rats (*n* = 20) were fed a high-fat diet (HFD) to induce metabolic dysregulation. After 12 weeks, OA was induced according to the Groove model. Two weeks after OA induction, rats were randomly divided into 2 groups and treated with 10 weekly, intra-articular injections of either rIL4-10 FP (*n* = 10) or phosphate buffered saline (PBS; *n* = 10). Possible antibody formation was evaluated using ELISA, cartilage degeneration and synovial inflammation were evaluated by histology and mechanical allodynia was evaluated using the von Frey test.

**Results:**

Intra-articular injections with rIL4-10 FP significantly reduced cartilage degeneration (*P* = 0.042) and decreased mechanical allodynia (*P* < 0.001) compared with PBS. Only mild synovial inflammation was found (nonsignificant), limiting detection of putative anti-inflammatory effects. Multiple injections of rIL4-10 FP did not induce antibodies against rIL4-10 FP.

**Conclusion:**

rIL4-10 FP showed chondroprotective and analgesic activity in a rat OA model with moderate cartilage damage, mild synovial inflammation, and pain. Future studies will need to address whether less frequent intra-articular injections, for example, with formulations with increased residence time, would also lead to DMOAD activity.

## Introduction

Osteoarthritis (OA) is the most prevalent chronic degenerative joint disease, predominantly characterized by cartilage damage and pain.^1^ Unfortunately, a disease-modifying OA drug (DMOAD) is still not available. The U.S. Food and Drug Administration and the European Medicines Agency demand a DMOAD to combine chondroprotective and analgesic effects in one molecule.^2,3^

Both interleukin 4 (IL-4) and IL-10 are immune modulatory cytokines that also act on different OA pathways.^4^ Besides its anti-inflammatory effects,^5^ IL-4 reduces cytokine-induced cartilage proteoglycan degradation in bovine cartilage explants.^6^ Likewise, IL-10 administered before or after axial compression protected against injury-induced apoptosis and extracellular matrix degradation *in vitro*.^7,8^ Both cytokines have immune modulatory effects, but IL-4 and IL-10 act differently,^9^ and possibly synergize in their immunoregulatory activity. IL-4 increases degradation of pro-inflammatory cytokine mRNA, whereas IL-10 inhibits nuclear factor κB and with that the transcription.^10^ Moreover, by combining the 2 cytokines, potential pro-inflammatory effects of IL-10 can be counteracted by IL-4.^10,11^ Combining both suppressed macroscopic signs of inflammation, reduced cellular infiltrates in synovial tissue, and protected against cartilage destruction, better than each of the cytokines alone, in a model of collagen-induced arthritis in mice.^12^ In hemophilic arthropathy, a joint disease with clear degenerative and inflammatory characteristics, the combination of IL-4 and IL-10 protected against this blood-induced cartilage damage.^13^

To combine the effects of both cytokines in a single molecule and increase bioavailability, a fusion protein of IL-4 and IL-10, IL4-10 FP, has been developed.^11^ IL4-10 FP inhibits pain in a mouse model for persistent inflammatory pain through inhibition of spinal neuroinflammation and inhibiting sensory neurons.^14,15^ In addition, IL4-10 FP has chondroprotective and anti-inflammatory effects in *in vitro* and *in vivo* models for hemophilic arthropathy^16^ as well as in human *in vitro* OA models.^17^

In order to achieve a high concentration in the joint and ensure maximal penetration into the joint issues while minimizing systemic side effects, the IL4-10 FP is specifically developed for intra-articular application.^18^ The chondroprotective and analgesic effects of IL4-10 FP were previously confirmed with intra-articular injections of canine IL4-10 FP in the canine OA Groove model.^19^

To test the IL4-10 FP in a rodent model of OA, the rat Groove model was used.^20^ This model of mechanically induced cartilage damage is mild and allows for tissue repair as there is no permanent trigger for joint damage (as, e.g., in instability or chemical induced models). When this damage is induced in rats that are metabolically dysregulated with a high-fat diet (HFD), joint damage increases,^21^ widening the window to evaluate DMOAD activity.

In the present study, rat IL4-10 FP (rIL4-10 FP) was developed and its DMOAD activity was tested on repeated intra-articular injections in the rat OA Groove model, in rats on HFD, to verify the results found in the canine OA Groove model. Using a rat model makes it possible to increase the study group. Besides, in contrast to previous studies, the model used here reflects a more age/obesity-associated, established/late-stage OA, making it more translatable to the human conditions.^21,22^

## Methods

### Study Design

Male Wistar rats (Charles-River, Sulzfeld, Germany; *n* = 20), 12 weeks old, were housed 2 per cage under 12:12 hour light-dark cycle, with access to high-fat food pallets (HFD; D12492i, Research Diets Inc., New Brunswick, NJ, USA) and tap water *ad libitum*. After 12 weeks, Groove surgery (OA induction) was performed in the right knee joint of each rat.^20^ In short, under general anesthesia, 5 longitudinal grooves of 150 to 180 µm depth were made in both femoral condyles, without damaging the underlying subchondral bone. Sham surgery was performed in the left knee joint of each rat, to serve as internal control. Analgesia (buprenorphine 10 to 50 µg/kg) was administered to all animals during the first 24 hours after surgery. Rats were allowed to move freely immediately after surgery.

**Figure 1.**
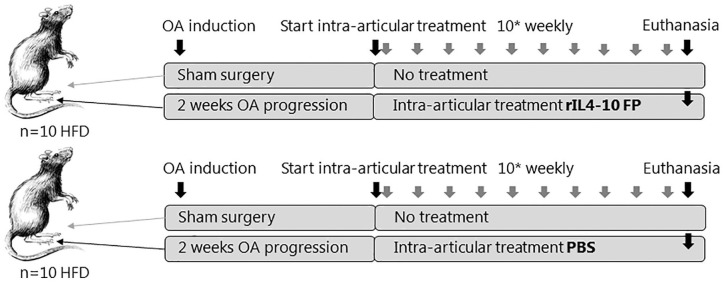
Experimental setup. HFD, 24 weeks old, 12 weeks high-fat diet male Wistar rats; OA, osteoarthritis; PBS, phosphate buffered saline.

Subsequently, 2 weeks after OA development, the rats were randomly divided into 2 groups of 10 rats each to receive 10 weekly intra-articular injections of either 0.5 µg in 25 µL rIL4-10 FP or 25 µL phosphate buffered saline (PBS) in the right, OA affected, knee joint, under general anesthesia. The concentration used is comparable to the concentration used in the canine study 10 µg in 500 µL. The sham operated knee joints were left untouched. After ten weeks with weekly intra-articular injections, rats were euthanized and tissue samples were harvested (**Fig. 1**). Blood samples of all rats in the rIL4-10 FP group were obtained at surgery, to determine baseline values, 3 weeks after start of intra-articular injections, and at the end of the study, to check for immunoglobulin G (IgG) antibody formation against the rIL4-10 FP. The study was approved by the Utrecht University Medical Ethical Committee for animal studies (AVD115002016688) and were fully compliant with ARRIVE guidelines.

### Production and Characterization of Rat IL4-10 FP

Production and characterization of rIL4-10 FP are largely done using the same procedures as published previously by this group for cIL4-10 FP.^19^

#### Transfection and Cell Culture

rIL4-10 FP was produced by transient transfection of HEK293F cells with a pcDNA3.1-neo expression vector (Invitrogen, Carlsbad, CA, USA) containing a dual CMV (cytomegalovirus) promoter. The vector contained 2 transgenes: cDNA coding for rIL4-10 and cDNA coding β-galactosidealpha-2,3-sialyltransferase to optimize glycan capping with sialic acid. To enable purification, a hexa-histidine affinity tag was cloned on the N-terminus of rIL4-10 FP. Cells were cultured in GIBCO FreeStyle 293 Expression Medium (Invitrogen). The medium contained no serum or antibiotics. Cells were grown in flasks on a shaker platform in humidified, 5% CO_2_ cell culture incubator at 37 °C. Cells were split 3 to 4 times prior to transfection and they were transfected at the cell viability of 90% and 1 million cells/mL cell density. The transfection reagent used was 293fectin (Invitrogen). Culture supernatant was harvested 72 hours after transfection.

#### Protein Purification

rIL4-10 FP was purified from culture medium via His-tag using Ni-NTA agarose (Qiagen, Hilden, Germany) according to manufacturer’s protocol. In short, protein was purified under native conditions with equilibration buffer (50 mM Na_2_HPO_4_, 0.3 M NaCl, 10 mM imidazole) and elution buffer (50 mM Na_2_HPO_4_, 0.3 M NaCl, 250 mM imidazole). Purified protein was dialyzed overnight against 2 L of PBS (pH 7.4), sterile filtered, and stored at −80 °C until use. Purity of rIL4-10 FP batches was evaluated by Coomassie-stained 12% SDS-PAGE (sodium dodecyl sulfate–polyacrylamide gel eletrophoresis) gel and HP-SEC (high-performance size exclusion chromatography) analysis.

#### Bioactivity Assay

The bioactivity of purified rIL4-10 FP was evaluated *in vitro* in a rat whole blood assay. Heparinized rat blood obtained on the day of surgery was diluted 1:10 in RPMI1640 medium, supplemented with 1% penicillin/streptomycin. Lipopolysaccharide (LPS) was added at 100 ng/mL final concentration. rIL4-10 FP as well as controls, recombinant rIL-4, and rIL-10, were simultaneously added and titrated in equal molar ratio’s, ranging from 0.001 to 3 nM. After 18-hour incubation at 37 °C, 5% CO_2_, rat tumor necrosis factor-α (TNFα) was measured in culture supernatants. The inhibition of inflammatory response by cytokines (rIL4-10 FP, rIL-4 or rIL-10) was calculated according to the formula: %inhibition = (1 − (A − B)/(C − B)) × 100, where A = TNFα levels in LPS-stimulated cultures treated with cytokines, B = TNFα levels in unstimulated culture, and C = TNFα levels in LPS-stimulated culture.

#### SDS-PAGE

Samples were diluted 1:1 in 2× Laemmli sample buffer (Bio-Rad, Hercules, CA, USA) containing 100 mM dl-dithiothreitol (Sigma-Aldrich, St. Louis, MO, USA), incubated 10 minutes at 95 °C, and loaded on a 12% polyacrylamide gel (Mini-PROTEAN-TGX; Bio-Rad). Electrophoresis was performed at 150 V for 1.5h, with reducing conditions (Tris/glycine/SDS buffer; Bio-Rad). Protein bands in the gel were visualized by Instant Blue protein stain (Expedeon, Heidelberg, Germany).

#### Western Blotting

After electrophoresis, proteins were transferred to a nitrocellulose membrane (Trans-Blot Turbo system, Bio-Rad). After blotting, membranes were blocked in 5% milk (Elk; Campina, Zaltbommel, Netherlands) in PBS with 0.1% Tween-20 (PBST; Merck, Darmstadt, Germany) and thereafter incubated overnight with primary antibody (biotinylated goat anti-rat-IL4; BAF504 0.1 µg/mL, or biotinylated goat anti-rat-IL10; BAF519 0.1 µg/mL, R&D systems, Minneapolis, MN, USA) in PBST containing 1% milk. Membranes were subsequently incubated with poly-HRP (poly–horse radish peroxidase) (Sanguine, Sherman Oaks, CA, USA) for 30 minutes at room temperature. To visualize the bands ECL Western Blotting Substrate was added according to the manufacturer’s protocol (Pierce, Thermo Fisher Scientific, Waltham, MA, USA).

#### Evaluation of Immunogenicity of rIL4-10 FP

Immunogenicity of rIL4-10 FP was evaluated by measuring the antibody titer in rat sera using enzyme-linked immunosorbent assay (ELISA kit DY501, R&D systems). Wells were coated with 1 µg/mL of rIL4-10 FP and were allowed to react with appropriately diluted rat sera, followed by incubation with HRP-labeled goat anti-rat IgGs (Sanguine) and then TMB substrate solution. Antibody titer was determined using the endpoint dilution. Serial dilutions of sera from rats treated with PBS were used to define the background OD.

### Mechanical Allodynia Measurement

Mechanical allodynia was assessed in 6 randomly selected rats of the 10 rats in each group on a power calculation with an effect size of 2.667, correction for multiple testing, and a power of 90%. Assessment was performed 24 hours before, and 24 hours after each intra-articular injection of either rIL4-10 FP or PBS. The experimenter was blinded to treatment. Rats were acclimatized to the specific set-up three times before testing and placed in enclosures on an elevated wire mesh floorwhere mechanical allodynia was assessed by applying von Frey hairs (Stoelting, Wood Dale, IL, USA) to the plantar surface of both hind paws. The hair force was increased or decreased according to the response and the 50% paw withdrawal threshold (PWT) was calculated using the up-and-down method as previously described.^23^

### Histopathological Examination of the Knee Joint

At the end of the study, the joint degeneration of both knee joints was evaluated using the OsteoArthritis Research Society International (OARSI) histopathology score for rats.^24^ In short, knee joint were fixed in formalin and subsequently embedded in paraffin. Coronal plane sections of 5 µm thickness were made at 100 µm intervals. Safranin-O (Saf-O) staining was used to assess joint histopathology. The total OARSI score is based on the sum of the following subsections: cartilage matrix loss width (0-2), cartilage degeneration (0-5), cartilage degeneration width (0-4), osteophytes (0-4), calcified cartilage and subchondral bone damage (0-5), and synovial membrane inflammation (0-4).Sections were scored in random order by 2 experienced observers, blinded for treatment. The surgically applied grooves were not taken into account during scoring.

### Statistical Analysis

To evaluate the analgesic effects of rIL4-10 FP a linear mixed model was used to account for the repeated PWT over time within subjects. The difference in PWT between the treated paw and control paw was used as outcome in this analysis and a random intercept at the level of subject was used. Treatment week, injection time (pre- or postinjection) and treatment groups (PBS or rIL4-10 FP) were used as fixed independent variables. To test whether the effect of injections over time (pre- vs. postinjection) was different between PBS or rIL4-10 FP treated subjects, the interaction between group and injection times was tested. Separate analysis within treatment groups were also performed, and the stability of the treatment effect over time was tested.

Histological data are presented as mean values with standard deviation (SD). Changes between OA joints and control joints were calculated. In 3 rats of each group, the experimental joint as well as the control joint could not be evaluated due to technical reasons (incorrect views) and as such not reliable assessed. The remaining material was insufficient to repeat the procedure. One additional control joint could not be scored in each group. The value for this control joint was imputed by taking the mean of the other 6 control joints. Outliers defined by values higher or lower than mean ± 2*SD were excluded (*n* = 1 for each group). Mann-Whitney *U* test was used to compare change scores between rIL4-10 FP and PBS groups.

## Results

### rIL4-10 FP Characteristics

**Figure 2.**
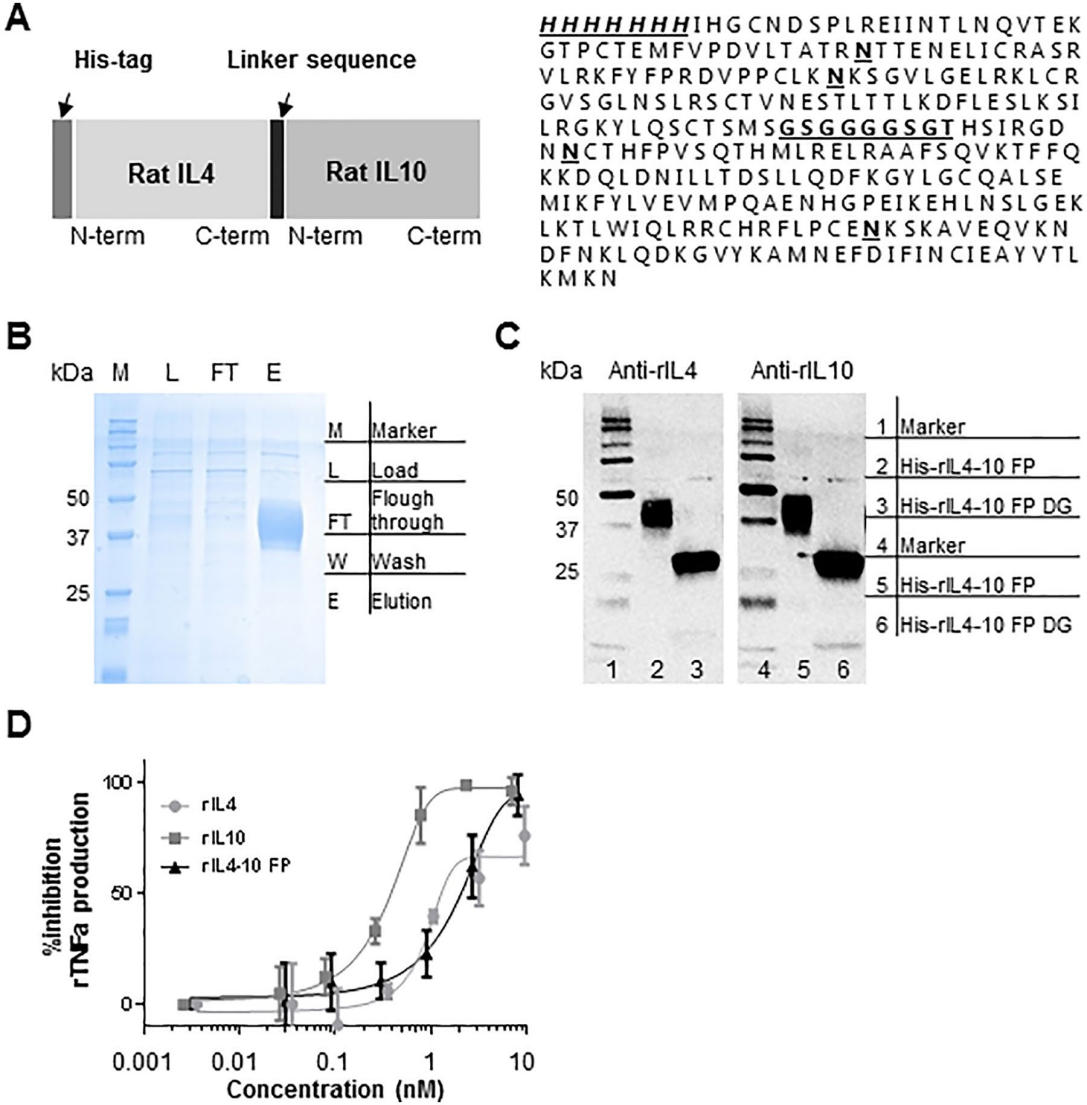
Molecular characterization of the rIL4-10 FP (rat interleukin-4–interleukin-10 fusion protein). Production and characterization of rIL4-10 FP are largely done according to the procedures as published previously by this group for cIL4-10 FP.^19^ (**A**) Schematic overview of the rIL4-10 FP and its amino acid sequence. N-terminal His-tag and linker sequence are indicated in bold italic. Potential N-linked glycosylation sites are indicated in bold. (**B**) Coomassie stained SDS gel of Ni-NTA protein purification steps. M, marker; L, load; FT, flow-through; W, wash; E, elution. (C) Western blot analysis of purified rIL4-10 FP (untreated and deglycosylated). (**D**) Bioactivity assay performed in rat whole blood culture. Blood was collected for *in vitro* testing at day of surgery, independent of treatment. Activity of rIL4-10 FP was evaluated *in vitro* according to its ability to inhibit TNFα secretion as a response to lipopolysaccharide stimulation.

A schematic representation of the rIL4-10 FP and its amino acid sequence, including the linker sequence and 4 predicted glycosylation sites are depicted in **Figure 2A**. Protein was produced by transient transfection of HEK293F cells and purified from culture supernatant by Ni-NTA affinity chromatography. Purified rIL4-10 FP was observed on Coomassie stained SDS gel as a smear composed of multiple protein bands with a molecular mass of 35 to 45kDa (**Fig. 2B**). Smeared protein bands were also detected on a Western blot by anti-IL4 and anti-IL10 antibodies, while on deglycosylation with PNGaseF only one sharp protein band of 30 kDa was detected (**Fig. 2C**), indicating that the multiple bands correspond to different glycoforms of rIL4-10 FP. The bioactivity of rIL4-10 FP was evaluated *in vitro* by its ability to inhibit TNFα production in an LPS-stimulated rat whole blood culture (**Fig. 2D**). The full inhibition of TNFα production was achieved with rIL4-10 FP at 8.3 nM, while IL-10 fully inhibited LPS-induced TNFα at 0.8 nM.Thus, *in vitro* rIL4-10 FP activity is approximately 10-fold lower compared with IL10.

### Mechanical Allodynia

**Figure 3.**
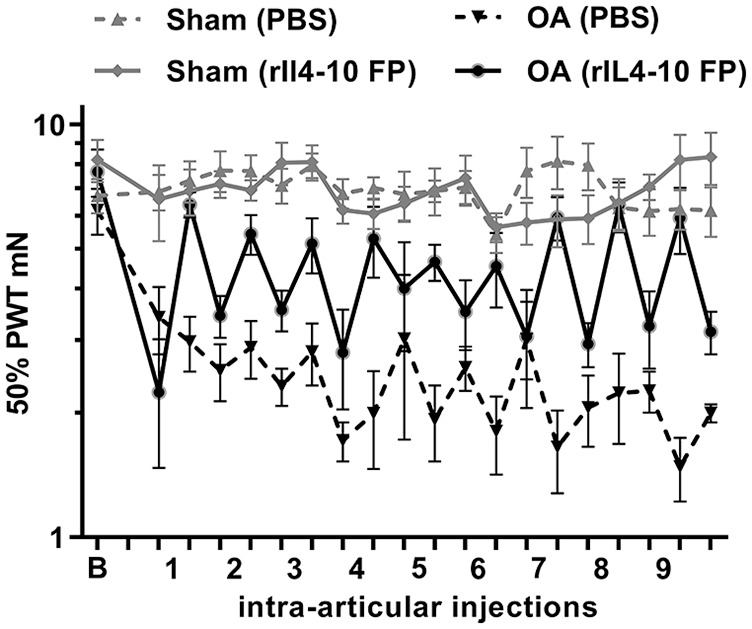
Effects of rIL4-10 FP (rat interleukin-4–interleukin-10 fusion protein) on mechanical allodynia. The difference in 50% paw withdrawal thresholds (PWT) between osteoarthritis (OA) paws and control paws reduces 24 hours after intra-articular injection with rIL4-10 FP compared with 24 hours before intra-articular injection.

The PWT in experimental and contralateral paws in both groups over time are presented in **Figure 3**. The intercept of the model indicating average difference in baseline PWT (i.e., before intra-articular treatment) between control and experimental paws was −4.46 mN (95% CI −5.56 to −3.36, *P* < 0.001) indicating that pain hypersensitivity developed over time in the OA Groove model.

In the linear mixed model, a statistically significant interaction was found between treatment group and the effect of intra-articular injections (*P* < 0.001) indicating that the effect of rIL4-10 FP injections on PWT is beneficial over PBS injections with an average difference of 2.68 mN (95% CI 1.50-3.83).

Analyzing only rats treated with rIL4-10 FP injections showed that, on average, an intra-articular injection with rIL4-10 FP reduced the difference in PWT between OA and control paw after injection (compared with before injection) with 2.45 (95% CI 1.52-3.37, *P* < 0.001, **Table 1**). This effect was not found to be different over time (*P* = 0.779). Analyzing only PBS-injected rats showed no statistically significant effects on PWT.

**Table 1.** Effects of rIL4-10 FP on Pain Using a Linear Mixed Model Analysis.^a^

Independent Variable	Coefficient	*P*	95% CI	
			Lower Bound	Upper Bound
Intercept	–3.82	<0.001	–7.86	0.22
Injection	2.45	<0.001	1.52	3.37
Week	0.02	0.803	–0.15	0.19

rIL4-10 FP = rat interleukin-4–interleukin-10 fusion protein.

a Pain was measured by determining the 50% pain withdrawal thresholds (PWT) using von Frey test. The intercept indicates the average difference in baseline PWT between control and experimental (Grooved) paws before intra-articular treatment.

### Joint Histopathology

Local cartilage damage induced according to the Groove model resulted in increased joint degeneration 12 weeks postsurgery (2 weeks OA development followed by 10 weeks intra-articular injections) compared with the sham operated control knee joints in both groups (OARSI histopathology score 9.8 ± 2.2 vs. 3.0 ± 1.8 and 6.8 *±* 1.9 vs. 3.0 ± 1.1 for PBS and rIL4-10 FP, respectively). These results are in line with previous published data using this model.^21^

**Figure 4.**
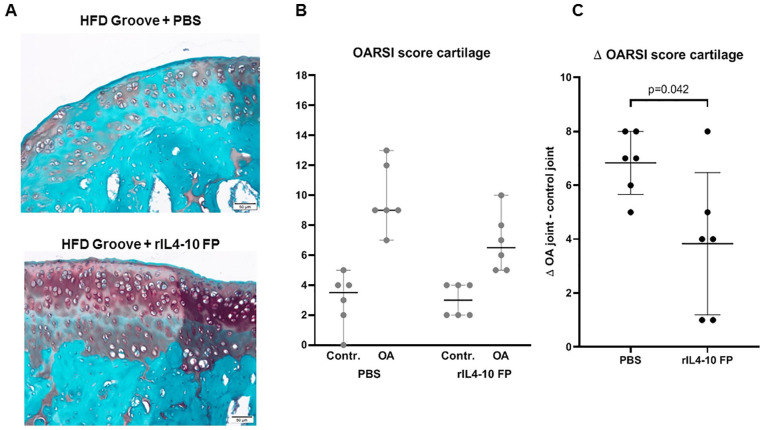
Cartilage degeneration. (**A**) Pictures of safranin-O stained joints of PBS injected and rIL4-10 FP injected joints. (**B**) OARSI scores for cartilage and bone damage of sham control and treated OA joints 10 weeks after treatment for the PBS and the rIL4-10 FP injected animals. Average scores (median values with 95% CI) are provided for each group. Maximum score is 20 points. (**C**) Differences in cartilage degeneration between sham control joint and treated OA joints for each individual animal. Average scores (median values with 95% CI interval) are provided for each group. PBS = phosphate buffered saline; rIL4-10 FP = rat interleukin-4–interleukin-10 fusion protein; OARSI = Osteoarthritis Research Society International; OA = osteoarthritis; HFD = high-fat diet.

The increase in cartilage damage after OA induction was less in the rIL4-10 FP group compared to the PBS injected group (Δ OARSI score +6.8 ± 1.2 vs. + 3.8 ± 2.6 in PBS and rIL4-10 FP injected group, respectively, *P* = 0.042, **Fig. 4**).

**Figure 5.**
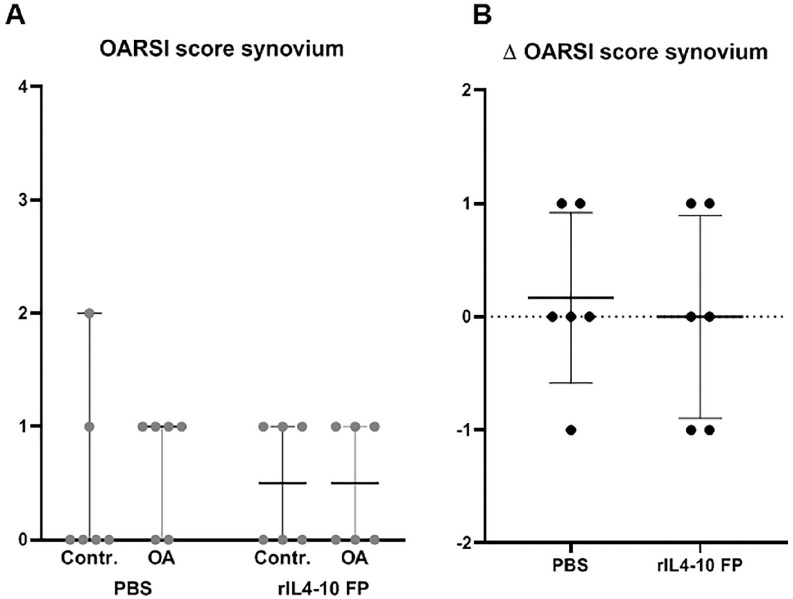
Synovial inflammation. (**A**) OARSI synovial inflammation scores of sham control and treated OA joints 10 weeks after treatment for the PBS and the rIL4-10 FP injected groups. Average scores (median values with 95% CI) are provided for each group. Maximum score is 4 points. Differences in synovial inflammation between sham control joint and treated OA joints. Average scores (median values with 95% CI) are provided for each group. PBS = phosphate buffered saline; rIL4-10 FP = rat interleukin-4–interleukin-10 fusion protein; OARSI, Osteoarthritis Research Society International; OA = osteoarthritis; IgG = immunoglobulin G.

Only mild synovial inflammation was found 0.5 ± 0.8 versus 0.7 ± 0.5 for sham-operated versus PBS-injected joints (nonsignificant), and 0.5 ± 0.5 versus 0.5 ± 0.5 for sham-operated versus rIL4-10 FP injected joints (nonsignificant), preventing evaluation of anti-inflammatory effects (**Fig. 5**). The change in inflammation after OA induction in the rIL4-10FP treated group was not different between both groups (0.1 ± 1.0 vs. 0.5 ± 0.9, for rIL4-10 FP and PBS, respectively).

### Immunogenicity of rIL4-10 FP

**Figure 6.**
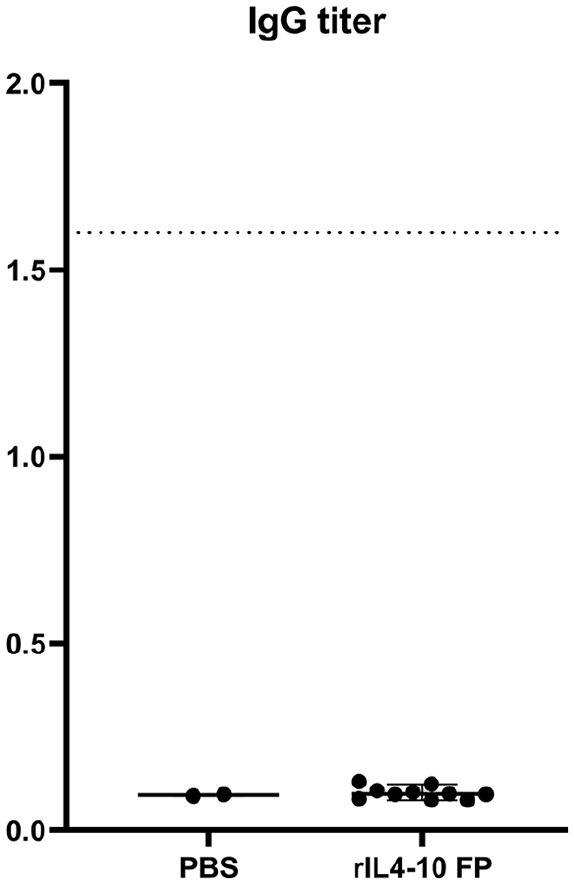
Immunogenicity of rIL4-10 FP. IgG antibody titers in sera of PBS-injected rats (*n* = 2) and rIL4-10 FP injected rats (*n* = 10). The dotted line represents the titer of 1.6 after an immunogenic response on human IL4-10 FP in the canine model. PBS = phosphate buffered saline; rIL4-10 FP = rat interleukin-4–interleukin-10 fusion protein; IgG = immunoglobulin G.

The IgG antibody titer found in sera of PBS-treated rats (*n* = 2) and rIL4-10 FP treated rats (*n* = 10) was 1/1000, corresponding to the background signal, indicating that rIL4-10 FP was not immunogenic in rats after 10 weekly intra-articular injections (**Fig. 6**).

## Discussion

This study for the first time shows the disease modifying effects of a species-specific fusion protein of IL-4 and IL-10 in a rat OA model. The concentration used in this rat study (500 ng/25 µL) is comparable to the concentration used in the canine model (10 µg/500 µL).^19^ Intra-articular treatment started 2 weeks after OA induction. The OA status after 2 weeks is unknown but considered mild, therefore the described effects of rIL4-10 FP treatment possibly represent the prevention of (further) development of OA after mechanical cartilage damage, rather than actual treatment of more established OA. Clinical application of the fusion protein is also anticipated at a relative early stage of the disease, arresting the degenerative process.

In the canine Groove model, human IL4-10 FP led to IgG antibody titers of 1.6, whereas a species-specific canine IL4-10 FP did not lead to antibody formation.^19^ In the present study, the absence of IgG antibody formation in case of rIL4-10 FP injections in rats confirmed the nonimmunogenic nature of a species-specific IL4-10 FP. Repeated intra-articular injection with rIL4-10 FP restored the by the OA model increased mechanical allodynia to values comparable to the control paws. Additionally, the histologically observed joint damage, developed over a 12-week period in the OA joints, was significantly less in the rIL4-10 FP injected group as compared with the PBS-injected group. The histopathology clearly shows severe progression of tissue damage in the PBS-treated animals, with the OARSI histopathology scores reaching high values on average, as compared with the fusion protein–treated animals, with lower scores on average and clearly more healthy joint tissue. Analgesic and chondroprotective effects are two important features of a DMOAD. The potential of IL4-10 FP as a DMOAD is further supported as these rodent data corroborate comparable DMOAD effects of canine IL4-10 FP in the canine Groove model of OA^19^ and chondroprotective effects of human IL4-10 FP in human OA cartilage explants and pain relief in a dog model.^17^ Moreover, similar analgesic and chondroprotective effects of human IL4-10 FP have also been observed in a mouse model of hemophilic arthropathy.^16^

Due to an absence of a significant inflammatory component in this rat model, an anti-inflammatory effect of rIL4-10 FP could not be confirmed. However, previous studies demonstrated anti-inflammatory effects of the IL4-10 FP.^13,16,17^

The preclinical evaluation of DMOADs is frequently performed by treating prophylactically or early in the OA process, immediately after OA induction (mostly posttraumatic OA), in young and normal-weight animals. This does not match the clinical OA population, which is focused on age-related established/late-stage OA frequently associated with obesity.^22^ Thus, the OA target population and preclinical phenotype are often mismatched. In this study, a combination of HFD and Groove surgery was used as OA model. This combination results in a more clinically relevant model of OA.^21^ Therefore it was anticipated that this model is suitable to evaluate in a more translational approach the DMOAD activity of rIL4-10 FP.

Pain is the predominant symptom of OA and the reason why OA patients seek medical assistance.^1^ In this study a transient analgesic effect of intra-articular injections with a species specific IL4-10 FP was found. Pain was assessed in a limited number of rats (6 per group), nevertheless, the results are in line with previous results, found in an *in vivo* canine model of OA.^19^

*In vitro*, the activity of rIL4-10 FP is 10-fold lower compared to the activity of solely IL10 (**Fig. 2D**). However, full inhibition is achieved by both, and combining IL-10 with IL-4 increases bioavailability and effectuates possible synergy between both cytokines.

The mechanisms by which intra-articular injected IL4-10 FP reduced mechanical hypersensitivity are not yet clear. However, recently we have shown that IL4-10 FP when injected intrathecally inhibits inflammatory pain through direct signaling to sensory neurons.^15^ Indeed various studies showed that cytokines, such as IL-10 and IL-4 may have direct effects on neurons and control their excitability.^25-29^ Although IL4-10 FP may have direct analgesic properties through direct effects on sensory neurons, subchondral bone osteoclasts and chondrocytes also contribute to OA pain^30,31^ and are known to express IL-4 and IL-10 receptors.^13,32,33^ Thus it is likely that the observed analgesic effect of IL4-10 FP is also mediated (in part) through indirect actions through osteoclast and chondrocytes or even other intermediate cells.

In general, rapid clearance from the joint cavity is a major drawback of intra-articular administration of drugs and requires repetitive injections after relatively short time periods. In a murine model for persistent inflammatory pain, intra-articular injection did not influence hyperalgesia at all, whereas intrathecal treatment with IL4-10 FP reduced pain for a time period of 2 to 4 days.^14^ Nevertheless, this is still too short for use in clinical practice. These results clearly show that despite the analgesic activity, more sustained analgesic effects on a single intra-articular injection, or other delivery routes, become key in future studies. Therefore, an important goal in the development of local OA treatment should be extending the duration of effects within the joint to enhance clinical relevance. A slow-release formulation of a hydrogel has previously been proposed with good results in animal studies.^34^

In conclusion, repeated (weekly) intra-articular injections of rIL4-10 FP in this rat OA model with OA development in a metabolic dysregulated background results in relief of OA induced pain and prevents/slows down joint damage. As such, DMOAD activity of IL4-10 FP in this model but also its activity in a dog *in vivo* model and human OA *in vitro* models warrant further research. In particular an improved understanding of the requirements for an increased duration of action will be needed to enable clinical development of this potential DMOAD.
